# β-Glucan Synthase Gene Overexpression and β-Glucans Overproduction in *Pleurotus ostreatus* Using Promoter Swapping

**DOI:** 10.1371/journal.pone.0061693

**Published:** 2013-04-24

**Authors:** Ran Chai, Cuiwei Qiu, Dongren Liu, Yuancheng Qi, Yuqian Gao, Jinwen Shen, Liyou Qiu

**Affiliations:** 1 College of Life Sciences, Henan Agricultural University, Zhengzhou, People's Republic of China; 2 Key Laboratory of Enzyme Engineering of Agricultural Microbiology, Ministry of Agriculture, Zhengzhou, People's Republic of China; University of California Riverside, United States of America

## Abstract

Mushroom β-glucans are potent immunological stimulators in medicine, but their productivities are very low. In this study, we successfully improved its production by promoter engineering in *Pleurotus ostreatus*. The promoter for β-1,3-glucan synthase gene (*GLS*) was replaced by the promoter of glyceraldehyde-3-phosphate dehydrogenase gene of *Aspergillus nidulans*. The homologous recombination fragment for swapping *GLS* promoter comprised five segments, which were fused by two rounds of combined touchdown PCR and overlap extension PCR (TD-OE PCR), and was introduced into *P. ostreatus* through PEG/CaCl_2_-mediated protoplast transformation. The transformants exhibited one to three fold higher transcription of *GLS* gene and produced 32% to 131% higher yield of β-glucans than the wild type. The polysaccharide yields had a significant positive correlation to the *GLS* gene expression. The infrared spectra of the polysaccharides all displayed the typical absorption peaks of β-glucans. This is the first report of successful swapping of promoters in filamentous fungi.

## Introduction

Mushroom β-glucans are the major structural constituents of the mushroom cell wall. They also provide antioxidant activity [1], [2] and can be used as immunological stimulators in medicine, such as antitumor, immunomodulating, antioxidant, radical scavenging, cardiovascular, antihypercholesterolemia, antiviral, antibacterial, antiparasitic, antifungal, detoxification, hepatoprotective, and antidiabetic effects [3]. Therefore, there has been growing popularity in developing mushroom β-glucans as drugs or dietary supplements and scientifically investigating their functions [4]. However, controlled by metabolic regulation, the production of β-glucans in mushroom is low. Furthermore, due to enzymatic degradation by glucanase activity during storage, the production from fruiting body or submerged fermentation liquid is only 20–50 mg/100 g dry matter of fruiting body [5] or 0.15–5.3 g/l ferment broth [6], [7]. Accordingly, it is of great value to improve β-glucans productivity in mushrooms by genetic engineering.

Most mushroom β-glucans with immunological stimulation are β-(1→6)-branched β-(1→3)-linked [8], and are synthesized by β-1,3-glucan synthase (GLS) (UDP-glucose 1,3-β-D-glucan 3-β-D-glucosyl transferase, EC 2.4.1.34). The GLS complex is composed of a catalytic subunit FKS and a regulatory subunit RHO. FKS is activated by RHO, a G protein, through GTP-dephosphorylation, and synthesizes the polymer of glucose monomers. RHO activity is regulated by a wall GDP-GTP exchange factor protein [9]. In fungi genomes, FKS and RHO genes are highly conserved and usually have only one or two copies [10], [11]. The information about the transcriptional regulation of *GLS* has been scant [11].

In yeast, GLS activity, both at transcriptional and enzymatic level, is stimulated by stress-inducing compounds present in the media [12]. A similar mechanism is also present in *Lentinula edodes* [13]–[15]. Nevertheless, the expression of the *GLS* genes in *Ustilago maydis*, a fungus causing smut disease on maize, was constitutive during its infection in maize and in response to ionic and osmotic stress [11].

Oyster mushroom *Pleurotus ostreatus* is a widely cultivated edible and medicinal mushroom in China and East Asia due to its short growth time, high adaptability and productivity. Its β-glucans demonstrated efficacy in promoting the survival of mice susceptible to bacterial infections [16], high SOD-like activity and antitumor activity [17]. In this study, we obtained the sequences of its only *GLS* gene and the promoter from *P. ostreatus* PC15 v2.0 in JGI Genome Portal (http://genome.jgi-psf.org). Using homologous recombination, the *GLS* gene promoter of *P. ostreatus* was replaced by the promoter of glyceraldehyde-3-phosphate dehydrogenase (*gpd*) gene of *Aspergillus nidulans*; the transformants displayed high expression of *GLS* gene and high production of β-glucans.

## Materials and Methods

### Strains and DNAs

*Pleurotus ostreatus* TD300, often used as a commercial cultivation strain in China, was obtained from Zhengzhou Composite Experiment station, China Edible Fungi Research System (Zhengzhou, China), and cultivated on PDA medium at 28°C for six days as described elsewhere [18].

**Figure 1:**
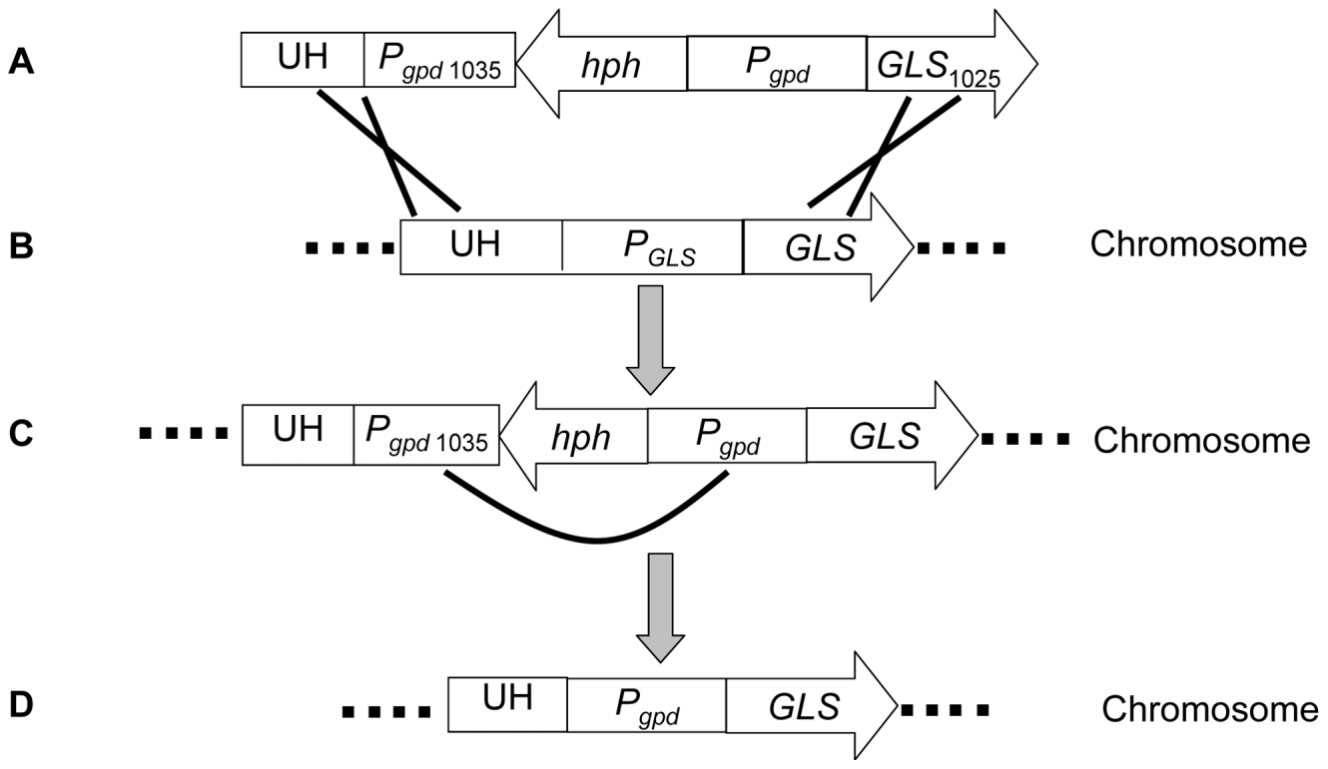
The outline of homologous recombination for promoter swapping. The homologous recombination fragment consisting of five DNA segments (A); the integration of the fragment into *P. ostreatus* chromosome via homologous recombination (B); the intramolecular homologous replacement of *P_gpd_*_ 1035_ by *P_gpd_* (C); the deletion of selection marker *hph* (D). UH: upstream homology sequence; *P_gpd_*_ 1035_: the partial 5′ end sequence of the *gpd* promoter in *Aspergillus nidulans*; *hph*: hygromycin B resistance gene (*hph*) of *E. coli* expression cassette; *P_gpd_*: the *gpd* promoter; *GLS*_1025_: the downstream homologous sequence to the 5′ end partial sequence of *GLS*.

The homologous recombination fragment for the *GLS* promoter swap comprised five segments: UH, P*_gpd_*_ 1035_, *hph*, P*_gpd_*, and *GLS*_1025_ (Fig. 1).

The 1,015 bp UH was the upstream homologous sequence which matched to the 3′ end partial sequence preceding the *GLS* promoter of *P. ostreatus*. It was cloned from the genomic DNA of *P. ostreatus* by PCR using primers UH-F and UF-R (Table 1), and its GeneBank accession number was JX889617. The primers were designed based on the genome sequence of *P. ostreatus* PC15 v2.0 in JGI Genome Portal (http://genome.jgi-psf.org). In the primer UF-R sequence, the last 15 nts (highlighted with underline) were complementary to the 5′ end sequence of *P_gpd_*_ 1035_.

**Table 1:** Primers used in this study.

Primers	Nucleotide sequence
UH-F	5′-TCCCCCGGGACTCGTTATCGTATTC-3′
UH-R	5′-TAGTCGTTGGCAGGCCAGTTTCCATATC-3′
P_gpd 1048_-F	5′-GATATGGAAACTGGCGAATTCCCTTGTATCT-3′
P_gpd 1048_-R	5′-GTAATCGTTGGCAGAAATCCTTACAGCTTG-3′
hph-F	5′-CAAGCTGTAAGGATTTCTGCCAACGACTA-3′
hph-R	5′-AGATACAAGGGAATTCATCAACATGCTACC-3′
P_gpd_-F	5′-GGTAGCATGTTGATGAATTCCCTTGTATCT-3′
P_gpd_-R	5′-GCTGTGTGAGTCCAATGGAG TATGGATGTAG-3′
GLS_1025_-F	5′-CTACATCCATACTCCATTGGACTCACACAGC-3′
GLS_1025_-R	5′-CGGGATCCAGTAAGTCTTGAAGAAAA-3′
hph-1	5′-GGAAGTGCTTGACATTGGGGAGTT-3′
hph-2	5′-TACTTCTACACAGCCATCGGTCCAG-3′
P_gpd_-1	5′-CAAGGTCGTTGCGTCAGTCC-3′
GLS-1	5′-CCAAGTCATAGGTGCAGAAGGA-3′
GLS-F	5′-AATTTGGATTCCAACGGGA-3′
GLS-R	5′-GGGCTATTGATCGCTTCTC-3′
AC-1	5′-GATAGAACCACCAATCCAAAC-3′
AC-2	5′-AAGTCATCACCATCGGTAACG-3′

The 1,035 bp *P_gpd_*_ 1035_ served as the upstream FLP recognition target (FRT) sequence; it was the partial 5′ end sequence of *P_gpd_*, the promoter of *gpd* gene in *Aspergillus nidulans*. *P_gpd_*_ 1035_ was generated from plasmid PAN7-1 by PCR using primers P*_gpd_*_ 1048_-F and P*_gpd_*_ 1048_-R (Table 1). The first 15 nts of P*_gpd_*_ 1048_-F and last 16 nts (with underline) of P*_gpd_*_ 1048_-R were complementary to the 3′ end sequence of UH and 5′ end sequence of *hph*, respectively. The plasmid pAN7-1 (kindly provided by Prof. van den Hondel, Leiden University, Netherlands) contains the hygromycin B resistance gene of *Escherichia coli* controlled by the *gpd* promoter and the transcription termination signal of tryptophan synthetase gene (*trp*C) from *A. nidulans* [19].

The 2,822 bp *hph* was hygromycin B resistance gene of *E. coli* expression cassette, and was amplified from plasmid PAN7-1 by PCR using primers hph-F and hph-R (Table 1). The first 16 nts of hph-F and last 14 nts (with underline) of hph-R were complementary to the 3′ end sequence of *P_gpd_*_ 1035_ and 5′ end sequence of *P_gpd_*, respectively.

The 2,206 bp *P_gpd_* was also amplified from plasmid PAN7-1 by PCR using primers P*_gpd_*-F and P*_gpd_*-R (Table 1). The first 14 nts of P*_gpd_*-F and last 17 nts (with underline) of P*_gpd_*-R were complementary to the 3′-end sequence of *hph* and 5′-end sequence of *GLS*_1025_, respectively.

**Figure 2:**
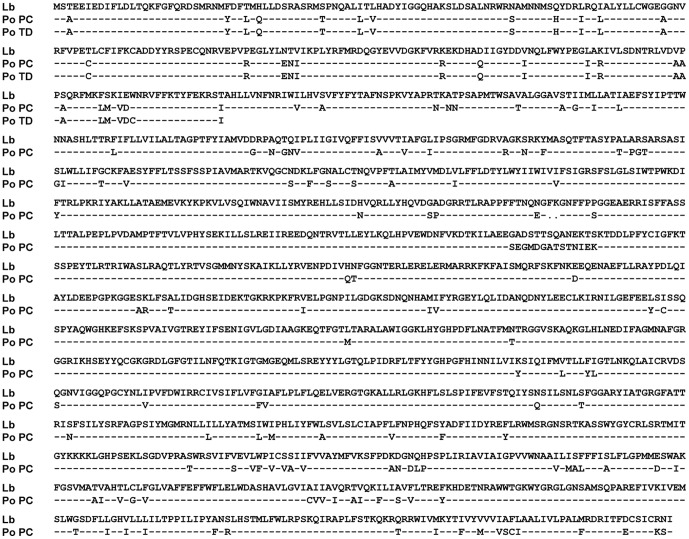
Comparison of predicted GLS amino acid sequences among *P. ostreatus* TD300, *Laccaria bicolor* S238N-H82, and *P. ostreatus* PC15 v2.0. **Lb**: GLS from *Laccaria bicolor* S238N-H82, GeneBank accession numbers is XM_001875351. **Po PC**: GLS from *P. ostreatus* PC15 v2.0, collected by visual inspection using the JGI Genome Portal for the *P. ostreatus* PC15 v2.0 genome (http://genome.jgi-psf.org); **Po TD**: GLS from *P. ostreatus* TD300, GeneBank accession numbers is JX889617.

The 1,025 bp *GLS*_1025_ was the downstream homologous sequence in the 5′ end partial sequence of *P. ostreatus GLS*. It was cloned from the genomic DNA of *P. ostreatus* by PCR using primers GLS_1025_-F and GLS_1025_-R (Table 1), and its accession number in GeneBank was JX889617. The primers were designed based on the only *GLS* gene sequence of *P. ostreatus* PC15 v2.0 collected by visual inspection using JGI Genome Portal (http://genome.jgi-psf.org). In the primer GLS_1025_-F sequence, the first 15 nts (with underline) was complementary to the 3′ end sequence of *P_gpd_*. The predicted amino acid sequences of *GLS*_1025_ from *P. ostreatus* TD300 was 99.6% and 86.3% identical to that of *P. ostreatus* PC15 v2.0 and *Laccaria bicolor* S238N-H82, and the *GLS* amino acid sequences from *P. ostreatus* PC15 v2.0 and *Laccaria bicolor* S238N-H82 shared 89.3% identity (Fig. 2).

### Touchdown-overlap extension PCR

Two rounds of combined touchdown PCR and overlap extension PCR (TD-OE PCR) were performed to fuse the above five long DNA segments.

The first round of TD-OE PCR was carried out for the fusion of the three segments: *hph*, *P_gpd_*, and *GLS*_1025_; it produced a long fusion segment *hPG*. This round includes two steps. In step I, 47 µL reaction solution contains 1 µL of each DNA segment at 0.5 µM, 5 µL of 10× PCR buffer, 8.0 µL of 2.5 mM of dNTP, and 0.5 µL of 5 U/µL LA Taq. Amplification started at 94°C for 40 sec; the annealing temperature of the reaction decreased from 61.5°C to a touchdown 57.5°C at the cooling rate of 0.5°C every cycle, followed by five cycles at 57.5°C, 4 min at 68°C, and 10 min at 72°C. In step II, the reaction solution from Step I was added to 1.0 µL of 0.1 mM hph-F and GLS_1025_-R separately, and 0.5 µL LATaq. PCR conditions are similar to that of step I: amplification: 94°C for 40 sec; annealation: 60°C to 56°C decreasing by 0.5°C per cycle, then 20 cycles at 56°C; 7 min at 68°C, and 10 min at 72°C. After completion, 5 µL of the PCR reaction aliquots were analyzed on 1% agarose gels stained with ethidium bromide.

The second round of TD-OE PCR was carried out to fuse the three segments, i.e. UH, *P_gpd_*_ 1035_, and *hPG*, to generate the homologous recombination fragment. The PCR procedure was similar to that in the first round except for the annealing temperature and primers. In step I, the annealing temperature decreased from 62°C to a touchdown 58°C; in step II, added primers were UH-F and GLS_1025_-R, the annealing temperature decreased from 58°C to 56°C.

### PEG/CaCl_2_-mediated protoplast transformation and transformant identification

Protoplasts preparation and PEG/CaCl_2_-mediated transformation of *P. ostreatus* TD 300 were performed as described previously [20], [21]. The introduced foreign DNA was the homologous recombination fragment for swapping *GLS* promoter. Protoplasts of *P. ostreatus* were suspended in MTC buffer at 10^6^ protoplasts/mL, and 100 µL suspensions were mixed with 10 µg the DNA fragment. Transformants were subcultured on PDA with or without hygromycin B. A full description of this method is given in the Extended Methods S1. To verify the replacement of *GLS* promoter by the introduced fragment, two PCR reactions were performed using the genomic DNA of the transformants as template, primers hph-1 and hph-2 for the amplification of the *hph* gene, and primers P*_gpd_*-1 and GLS-1 (Table 1) for amplifying the combination of *P_gpd_* and *GLS*. Both PCR conditions were 94°C for 1 min, 56°C for 1 min, and 72°C for 1 min in 30 cycles. 5 µL of the PCR reaction aliquots were analyzed on 1% agarose gels stained with ethidium bromide.

### Semi-quantitative RT-PCR

To analyze the *GLS* expression in transformants, semi-quantitative RT-PCR was carried out as described elsewhere [21] with slight modifications. Total RNA was extracted from transformants. Primers were GLS-F and GLS-R for reverse transcription and amplification of *GLS*, and AC-1 and AC-2 for reverse transcription and amplification of housekeeping gene β-actin. Reverse transcription of the mRNA was carried out at 42°C for 60 mins by using MMLV Reverse Transcriptase 1st-Strand cDNA Synthesis Kit (Epicentre, Madison, WI, USA). The PCR conditions were 94°C for 40 sec, 49°C for 40 sec, and 72°C for 1 min in 30 cycles.

5 µL of the PCR reaction aliquots was analyzed on 1% agarose gels stained with ethidium bromide. The electrophoresis bands of RT-PCR reaction were photographed and the density of each band was quantified using image analysis software, UVI band V. 97 (UVI Tech, Cambridge, UK).

### Analysis of β-glucan content and infrared spectrum

To measure the β-glucan yield of the transformants, the transformants were cultivated in PD broth (150 mL in a 500 mL flask) at 25°C for 12 d under 150 rpm shaking. The culture broth was extracted in 98°C water bath for 3 h and then filtered; the supernatant was concentrated 10-fold via evaporation. Four volume 95% ethanol was added and placed overnight, centrifuged at 5,000·g for 15 min. The precipitate was washed 2 times with 85% ethanol, then dissolved in hot water, and de-proteinized by Sevag method [22]. Equal volume of Sevag reagent (chloroform/butanol 4∶1, v/v) was added, vigorously shaken for 30 min, the mixture was centrifuged at 5,000·g for 10 min. The upper layer was separated and Sevag reagent was again added. This process was repeated 3 times. The polysaccharide content was measured using phenol-sulfuric acid method [23]. The polysaccharide characteristic was determined by infrared spectroscopy (Tensor 37, Bruker, Ettlingen, Germany).

### Statistical analysis

Statistical analysis was performed using standard ANOVA techniques. Comparisons among the means of three independent experiments were made using the least significant difference (LSD) test.

## Results

### Construction of the homologous recombination fragment for swapping GLS promoter

**Figure 3:**
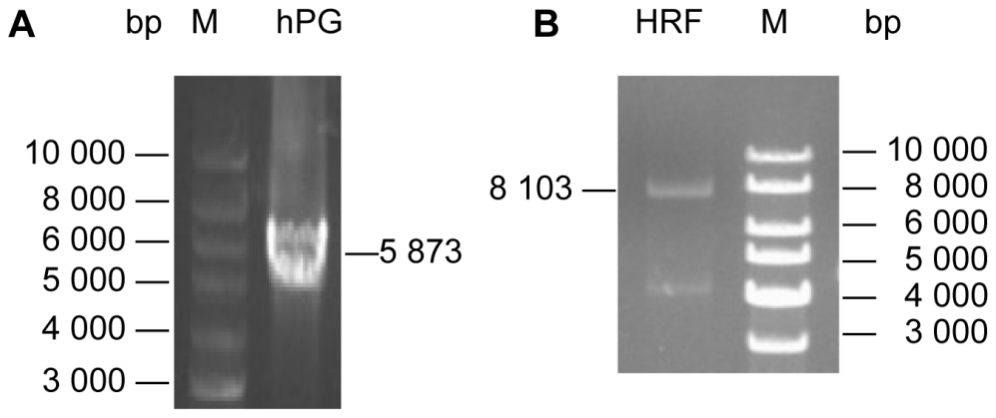
Agarose gel analysis of TD-OE PCR products for the construction of the homologous recombination fragment for promoter swapping. hPG: the fusion product of *hph*, *P_gpd_*, and *GLS*_1025_; HRF: the homologous recombination fragment which was the fusion product of UH, *P_gpd_*_ 1035_, and hPG.

The three segments of *hph*, *P_gpd_*, and *GLS*_1025_ were fused successfully, and the expected fusion product *hPG* (5,873 bp) was produced by only one round TD-OE PCR (Fig. 3A); *hPG* was subsequently fused with the two upstream segments UH and *P_gpd_*_ 1035_ in the second round of TD-OE PCR (Fig. 3B), and produced the homologous recombination fragment for swapping *GLS* promoter (8,103 bp). The sequencing result of the fusion product confirmed that all of the five segments were fused correctly in accordance with the design order.

### Identification of homologous recombination transformant by PCR

**Figure 4:**
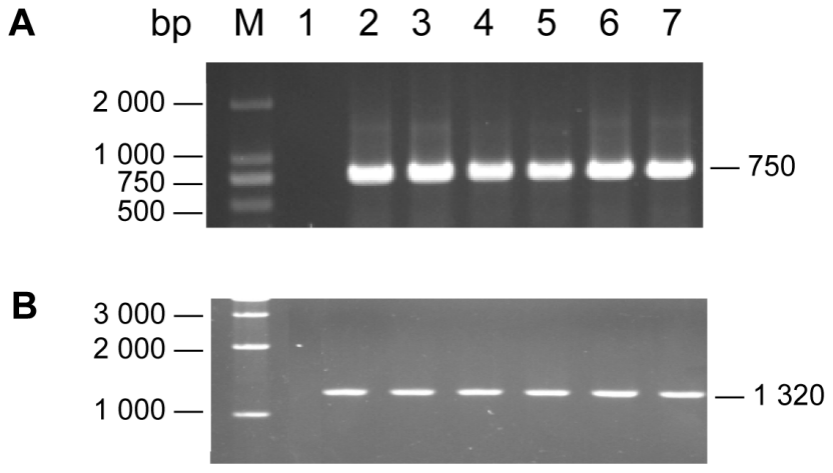
PCR for the identification of the recombinant *hph* and *P_gpd_* sequences in the transformants of *P. ostreatus*. PCR amplification on total DNA from the second generation transformants using primers hph-1 and hph-2 which defined a 750 bp sequence across the *hph* gene (A). PCR amplification on total DNA from the fifth generation transformants using primers P*_gpd_*-1 and GLS-1 which defined a 1,320 bp sequence spanning the *P_gpd_* and *GLS* gene located far from *GLS*_1025_ (B). Lane 1: WT; Lane 2–7: transformant A1, A4, A9, A15, A17, and A21.

The integration and the promoter swapping in the transformants were verified by PCR. Six transformants, A1, A4, A9, A15, A17, and A21, were randomly selected. The expected PCR amplifications were obtained from all the DNA samples of the second generation transformants using the primer pair hph-1 and hph-2, but not from the untransformed original strain (wild type) and the next generation transformants (Fig. 4A). The segment could be amplified from the DNA samples of the transformants from generation three to five by using the primer pair P*_gpd_*-1 and GLS-1, which was 1,320 bp in length and spanned the 3′ end of *P_gpd_* and the downstream of the 5′ end of *GLS*_1025_ (Fig. 4B). The result revealed that *hph* gene in the homologous recombination fragment was deleted by the homologous recombination between *P_gpd_*_ 1035_ and *P_gpd_* from the third generation of the transformants. In addition, the introduced *P_gpd_* replaced the *GLS* promoter and remained genetically stable.

### *GLS* gene overexpression and β-glucan overproduction of the transformants

**Figure 5:**
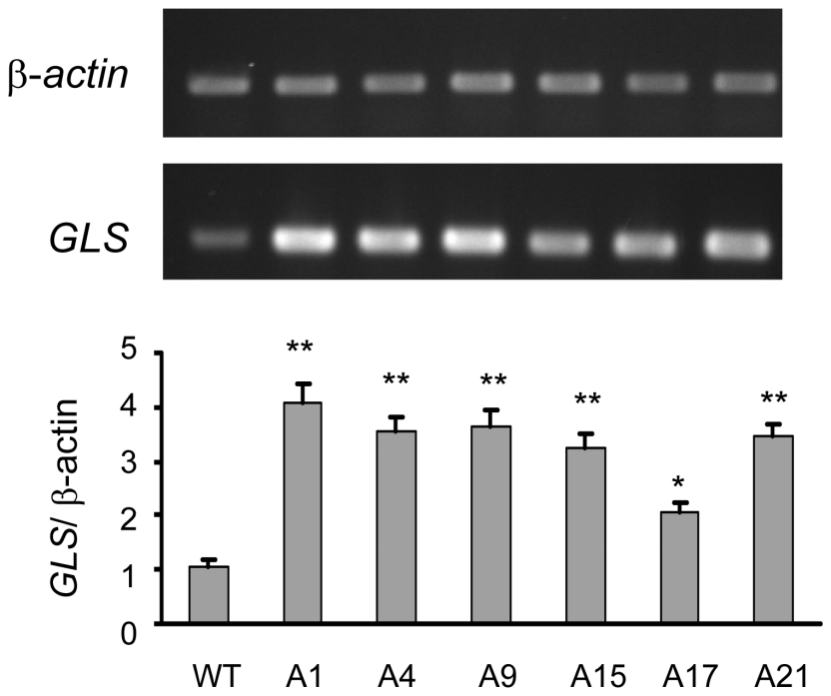
Semi-quantitative RT-PCR analysis of *GLS* mRNA in the fifth generation transformants. The amount of *GLS* mRNA, expressed as the ratio of densitometric measurement of the sample to the corresponding internal standard (β-*actin*), is shown in the upper panels. * *p*<0.05 comparing to WT; ** *p*<0.01 comparing to WT.

To determine whether *GLS* gene over-expressed after the swap of its promoter by *P_gpd_*, its mRNA expression level in the transformants was measured by semi-quantitative RT-PCR and was found to be two to four folds higher than that of wild type (Fig. 5).

**Figure 6:**
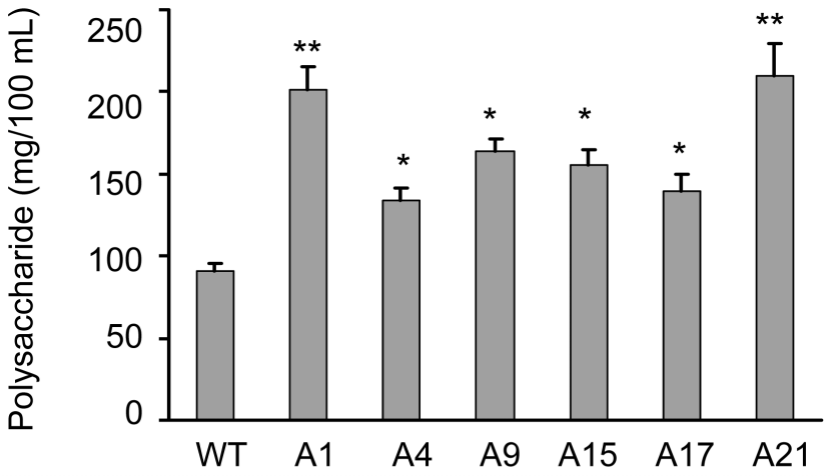
The polysaccharide yield of the fifth generation transformants. * *p*<0.05 comparing to WT; ** *p*<0.01 comparing to WT.

The polysaccharide yields of the six transformants were determined and they were 32% to 131% higher than that of wild type (Fig. 6); including the wild type, the yield had positive correlation to the *GLS* gene expression (*p*<0.05). The result indicated that the *GLS* gene expression may correspond with its enzymatic activity and protein level.

**Figure 7:**
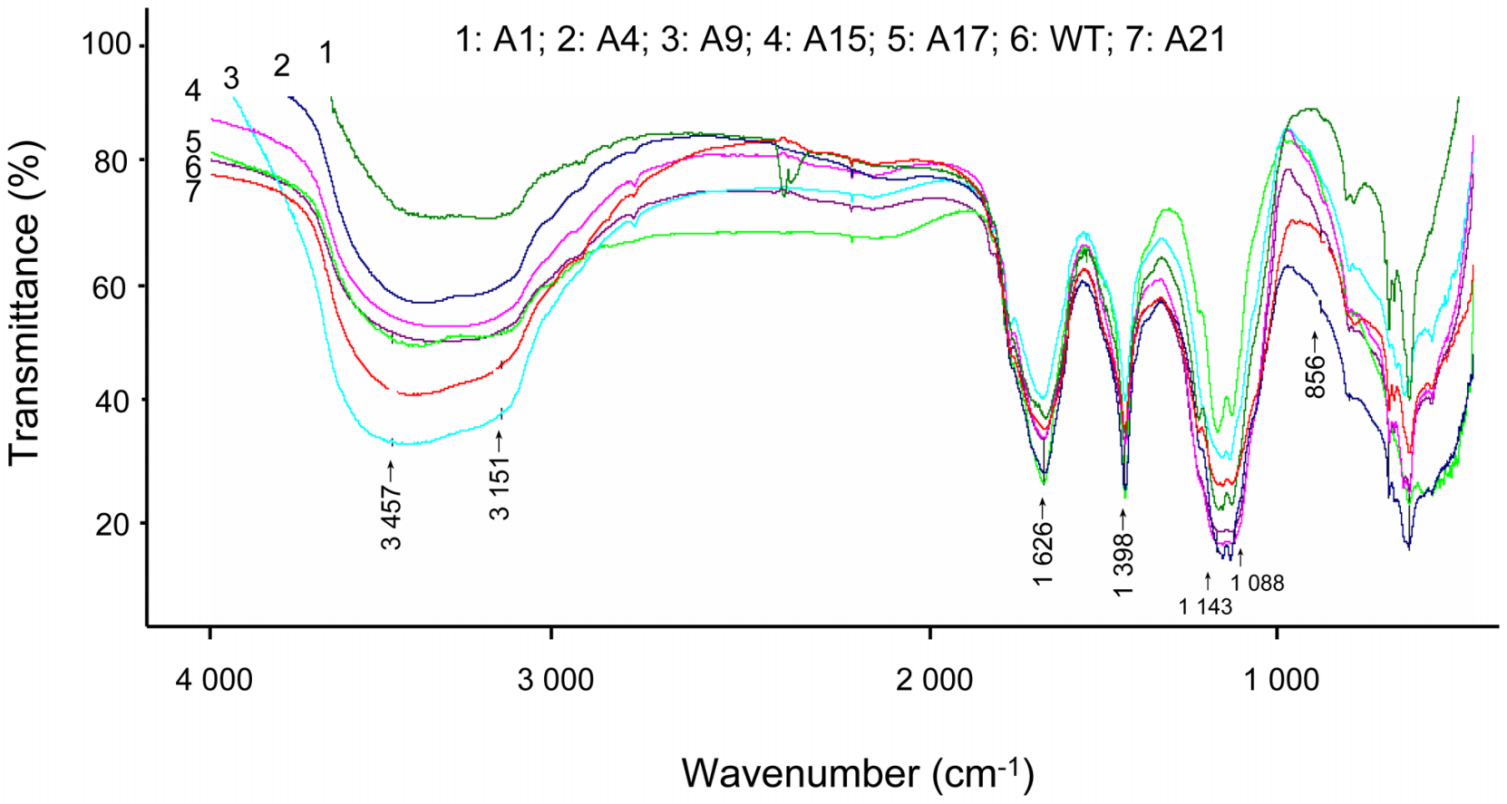
The infrared spectroscopy of the β-glucans produced by the fifth generation transformants.

The infrared spectra (IR) of the polysaccharides produced by the six transformants and wild type had the same characteristic with close resemblance to each other as shown in Fig. 7. The absorption peaks at 3,457 cm^−1^ were ascribed to hydroxyl stretching vibration of the polysaccharide; peaks at 3,151 cm^−1^ were CH (CH3, CH2, or CH) stretching vibration; those at 1,626 cm^−1^ attributed to C = O asymmetric stretching vibration [24]; those at 1,398 cm^−1^ were due to C-H variable-angle vibration; peaks at 1,143 to 1,088 cm^−1^ were characteristics of C-O-C asymmetric vibration of pyranose ring [25]; finally the bands at 856 cm^−1^ mapped to β-glucan [26], suggesting that β-glycosidic bond was present in all samples.

## Discussion

Promoter engineering is the principal strategy for metabolic engineering. It employs mutagenic endogenous promoters or introduced heterologous promoters to increase the expression of key pathway genes and maximize target production, and has been successfully applied to bacteria and yeast [27]–[30]. Nevertheless, the application of promoter engineering in filamentous fungi has not been reported. In this study, we introduced a heterologous promoter to increase the *GLS* expression and significantly improved β-glucan production.

In previous studies, the promoters for replacing native promoters could be constitutive or inducible [28]. Compared with constitutive promoters, inducible promoters tightly control their downstream gene to achieve high level expression, maximize protein production and reduce toxicity during growth phase [31]; but it is limited in practice due to inducer cost and cell hypersensitivity to inducer concentration [32]. *P_gpd_* is a constitutive promoter used across fungal species and provides high levels of constitutive gene expression [33], [34]; for example, the expression of genes under the control of *P_gpd_* was significantly higher than that by the commonly used alcohol oxidase 1 promoter (*P_AOX_*_1_) in methanol-grown cells of *Pichia pastoris* [35]. In this study, we employed *P_gpd_* from *A. nidulans* to swap the native promoter of *GLS* in *P. ostreatus*, and consequently *GLS* expression was improved by up to two folds and β-glucan production increased by up to 32% compared to the wild type strain. CaMV 35S is another constitutive promoter often used in filamentous fungi, which is considerably weaker than *P_gpd_* in *P. ostreatus* [21]. The other constitutive promoters used in filamentous fungi included *adhA*, *gdhA*, *oliC*, *pgkA*, etc [36], but the comparison among them has not been reported.

Construction of the homologous recombination fragment for swapping promoter requires five to six segments fusion [29], [37]. Multiple segments fusion is usually performed by overlap extension PCR (OE-PCR) [38], [39]. However, OE-PCR cannot fuse more than two DNA segments simultaneously [40]. Several modified OE-PCR procedures can fuse multiple segments at the same time, but require chimeric primers and high and close annealing temperatures in order to minimize mispriming [41], [42]. Touchdown PCR (TD-PCR) is an efficacious solution to reduce mispriming and rapidly optimize PCR to increase specificity, sensitivity, and yield [43], [44]. In this study, we combined TD-PCR and OE-PCR and used only two rounds to fuse the five segments. Our technique produced the homologous recombination fragment for swapping *GLS* promoter without sedulously adjusting the annealing temperature of primers. It showed that touchdown-overlap extension PCR (TD-OE PCR) was a fast and highly efficient method for promoter swapping and metabolic engineering.

### Conclusion

By our knowledge, this is the first report of successful swapping of promoters using TD-OE PCR in filamentous fungi through rapid construction of homologous recombination fragment. The polysaccharide yields of the transformants were 32% to 131% higher than that of wild type, and had significantly positive correlation to the *GLS* gene expression levels. TD-OE PCR, a novel procedure combining touchdown and overlap extension PCR, was carried out for the fusion of five segments to construct the homologous recombination fragment for swapping *GLS* promoter. Our study supports that TD-OE PCR was a fast and highly efficient method for promoter swapping and metabolic engineering.

## Supporting Information

Methods S1 **Extended Methods.**
(DOC)
