# Supplementary material for: β-Glucan Synthase Gene Overexpression and β-Glucans Overproduction in Pleurotus ostreatus Using Promoter Swapping
Source: PLoS One. 2013 Apr 24;8(4):e61693. doi: 10.1371/journal.pone.0061693 (PMC3634845; doi:10.1371/journal.pone.0061693)
Supplement: Methods S1 — Extended Methods. (DOC) [file pone.0061693.s001.doc]

**Methods S1**

**EXTENDED METHODS**

**PEG/CaCl_2_-mediated protoplast transformation**

Protoplasts of *P. ostreatus* TD300 were suspended in MTC buffer (0.6 M mannitol, 10 mM Tris-HCl, pH 7.5, 10 mM CaCl_2_) at 10^6^ protoplasts/ml, and 100 μl suspension was mixed with 10 μg the DNA fragment. The mixture was kept on ice for 5 min, after which 25 μL PEG solution (40% (w/v) PEG4000 (Merck), 10 mM Tris-HCl (pH 7.5), and 50 mM CaCl_2_) were added. Then it was kept on ice for 30 min, mixed gently with 0.6 ml the PEG solution, kept at room temperature for 30 min, and then 0.4 ml MTC buffer was added and kept at room temperature for 30 min. After adding 1 ml 0.6 M mannitol, the mixture was suspended in 4 ml RCM (0.2% tryptone, 0.2% yeast extract, 2% glucose, 0.05% MgS0_4_·7 H_2_0, 0.046% KH_2_P0_4_, 0.1% K_2_HP0_4_, 0.6 M mannitol) and incubated at 28℃ for 48 h. The suspension was centrifuged at 1 000∙g for 5 min, the pelleted protoplasts were spread on RCM agar plate prepared with 80 μg/ml hygromycin B and incubated at 28°C for 14 d.
